# Protective effects of melatonin and naringenin against acitretin induced hepatotoxicity via modulation of oxidative stress and inflammatory signaling

**DOI:** 10.1038/s41598-025-16740-9

**Published:** 2025-08-27

**Authors:** Samia S. Sokar, Sally E. Abu-Risha, Mahmoud Abdelrahman Alkabbani, Laila A. Ramadan, Alaa E. Elsisi

**Affiliations:** 1 Department of Pharmacology & Toxicology, Faculty of Pharmacy, Tanta University, Tanta, Egypt; 2 Department of Pharmacology & Toxicology, Faculty of Pharmacy, Egyptian Russian University, Cairo, 11829 Egypt

**Keywords:** Acitretin, Apoptosis, Hepatotoxicity, Inflammation, Melatonin, Naringenin, Gastroenterology, Medical research, Risk factors

## Abstract

Drug-induced liver injury (DILI) represents a major clinical challenge, often limiting the therapeutic use of agents such as acitretin, a second-generation retinoid prescribed for psoriasis. This study established and characterized a rat model of acitretin-induced hepatotoxicity, aiming to explore potential biomarkers and mechanisms of protection. Fifty male Sprague–Dawley rats were divided into five groups: control, acitretin, melatonin + acitretin, naringenin + acitretin, and combination treatment. Biochemical liver function tests, oxidative stress markers, inflammatory cytokines, histopathology, immunohistochemistry, and gene expression analyses were performed. Acitretin administration significantly elevated serum ALT, AST, ALP, LDH, and bilirubin levels, with concurrent reductions in serum albumin.Query Oxidative stress markers (MDA, nitrite) were increased, while antioxidant defenses (GSH, catalase) were compromised. Acitretin activated the HMGB1/TLR4/NF-κB pathway, elevated TNF-α and IL-6 levels, and triggered JAK/STAT3 signaling, contributing to inflammation, apoptosis via caspase-3 activation, and early fibrotic changes marked by TGF-β and MMP-9 upregulation. Treatment with melatonin and naringenin significantly mitigated these alterations, reducing oxidative stress, inflammation, apoptosis, and fibrogenesis. Notably, combination therapy provided superior hepatoprotection compared to individual treatments, suggesting a synergistic effect. These findings propose melatonin and naringenin as promising adjuncts to enhance the safety profile of acitretin therapy in clinical practice.

**Supplementary Information:**

The online version contains supplementary material available at 10.1038/s41598-025-16740-9.

## Introduction

Drug-induced liver injury (DILI) represents a significant cause of hepatic damage, resulting from the administration of pharmaceutical agents or other xenobiotics^1^. DILI is a leading cause of acute liver failure, frequently necessitating liver transplantation and resulting in significant mortality. Consequently, it presents a significant challenge in the development of drugs and in clinical practice^2^. DILI can be classified into two types: intrinsic and idiosyncratic. Intrinsic DILI is dose-dependent and can be anticipated based on the drug’s known toxicological profile. In contrast, idiosyncratic DILI is unpredictable, not dose-related, and occurs sporadically, making diagnosis and forecasting particularly challenging. Furthermore, idiosyncratic DILI is rarely replicated in animal models. Its pathogenesis is believed to involve multiple factors, including genetic predispositions and environmental influences such as the patient’s age, gender, underlying health conditions, and concurrent medication use^3–6^.

Acitretin, a second-generation retinoid approved by the FDA, is widely used for the treatment of psoriasis, either as a monotherapy or in combination with phototherapy, immunosuppressants, or biologics^7^. Besides its FDA-approved use in psoriasis, acitretin is employed off-label for other dermatological conditions such as Darier disease and for chemoprevention of non-melanoma skin cancers in transplant recipients^8^. Acitretin’s mechanism of action involves binding to retinoic acid receptors (RAR) and retinoid X receptors (RXR), modifying gene transcription resulting in anti-inflammatory and anti-proliferative effects, particularly in skin conditions like psoriasis^8–10^.

Despite its therapeutic efficacy, acitretin-induced liver damage has been extensively reported^11–17^. Clinical data demonstrate transitory increases in liver enzymes such as alanine transaminase (ALT), aspartate aminotransferase (AST), or lactate dehydrogenase (LDH) in 1 of every 3 of patients undergoing acitretin medication, with serious hepatotoxicity occurring in rare instances^18^. The first case of fulminant hepatic failure due to acitretin overdose has also been reported^19^. Acitretin induces mitochondrial dysfunction in hepatocytes, leading to a reduction in ATP production and increased mitochondrial susceptibility to calcium-induced permeability transition, which can trigger apoptosis and necrosis^13^. Moreover, acitretin-induced liver damage can manifest as cholestatic injury, warranting regular monitoring of both transaminases and cholestatic enzymes during treatment^14^.

Mitochondrial dysfunction in DILI leads to the overproduction of reactive oxygen species (ROS), which in turn causes oxidative stress and cellular damage. This process increases the presence of damage-associated molecular patterns (DAMPs), such as high mobility group box 1 (HMGB1), which trigger inflammatory pathways via Toll-like receptor 4 (TLR4)^20,21^. Activation of TLR4 via DAMPs triggers the MyD88-dependent pathway, resulting in the activation of the phosphoinositide 3-kinase (PI3K)/protein kinase B (Akt)/mammalian target of rapamycin (mTOR) pathway^22–25^. This cascade activates nuclear factor kappa B (NF-κB), facilitating the secretion of pro-inflammatory cytokines, such as tumor necrosis factor alpha (TNF-α), interleukin (IL)-1β, IL-6, IL-12, and cyclooxygenase-2 (COX-2)^26,27^. NF-κB is essential for initiating inflammatory responses in M1 macrophages, notably through the upregulation of matrix metalloproteinase-9 (MMP-9) production, which facilitates inflammation and tissue remodeling^28,29^.

The inflammatory process includes TNF-α-induced apoptosis of hepatocytes via activating caspase-3^30,31^. Moreover, IL-6 released by Kupffer cells or hepatocytes activates the JAK/STAT3 signaling pathway, which is pivotal to inflammation and stress responses^32^. Disruption of this pathway can result in immune deficiencies and malignancies^33^. In persistent liver damage, transforming growth factor-β (TGF-β) is pivotal in disease progression by activating stellate cells, resulting in fibrosis, cirrhosis, and ultimately hepatocellular carcinoma^34^. Elevated TGF-β levels are detected in cases of chronic liver injury, prompting stellate cells activation into fibrogenic myofibroblasts and death of hepatocytes, ultimately driving liver fibrosis^35,36^ Myofibroblasts contribute to fibrosis by producing excess collagen, while MMP-9 facilitates the breakdown of extracellular matrix components, further exacerbating liver damage^37,38^.

Melatonin, recognized for its function in regulating circadian rhythms^39^, has attracted considerable interest owing to its powerful hepatoprotective effects and its designation as the most potent endogenous antioxidant^40^. Its hepatoprotective effects have been demonstrated across various liver injury models, showing efficacy in conditions such as aflatoxin-induced liver injury^41^ and cadmium toxicity, where it regulates gut microbiota, intestinal FXR, and NLRP3 inflammasome pathways^42^. Melatonin also offers protection against sepsis-induced intestinal injury^43^, particulate matter (PM2.5)-induced liver fibrosis^44^, and ethanol-induced ferroptosis^45^ via mechanisms involving SIRT1, Nrf2 activation, and circadian regulation, respectively. Its hepatoprotective effects extend to chronic intermittent hypoxia-induced steatohepatitis^46^, hemorrhagic shock-induced liver injury by activating autophagy and the Akt-dependent HO-1 pathway^47^, and cholestatic liver injury^48^. A key mechanistic pathway for melatonin’s protective action is through inhibition of the HMGB1/TLR4/NF-κB signaling^41,49,50^, alongside downregulating TNF-α, caspase-3, IL-6, and TGF-β^48,51–53^, the same parameters are under investigation for possible mechanistic pathways of acitretin-induced liver injury.

Naringenin, a flavonoid naturally found in citrus fruits like grapefruits and sour oranges, has long been acknowledged for its pharmacological properties, including anti-inflammatory, antioxidant, anti-lipogenic, and hepatoprotective effects^54,55^. It has been thoroughly investigated for its capacity to alleviate liver damage induced by various agents, including taxol and acetaminophen^56,57^. Naringenin demonstrates hepatoprotective effects via various mechanisms, notably the inhibition of the TLR4/NF-κB pathway, thereby minimizing inflammation and oxidative stress in the liver^58,59^. It additionally downregulates pro-inflammatory cytokines such as TNF-α, IL-6, and TGF-β, inhibits apoptosis by modulating caspase-3 activity and Bax/Bcl-2 ratios, and augments antioxidant enzyme activities including SOD and reduced glutathione (GSH), thereby preserving redox equilibrium^55,56,59,60^. Naringenin also demonstrates anti-fibrotic properties by inhibiting PI3K/AKT signaling and suppressing MMP9 expression, thereby contributing to its protective effects against liver damage^59,61,62^.

Melatonin and naringenin have shown potential benefits in psoriasis management due to their anti-inflammatory, antioxidant, and immunomodulatory properties^63–67^. Clinical evidence supports the topical use of melatonin 5% cream, which significantly alleviated disease severity over 12 weeks with a favorable safety profile^63^, while oral melatonin at doses up to 5 mg/day has been safely used long-term for other indications^68,69^. Naringenin, although not yet approved for clinical use in psoriasis, demonstrated anti-inflammatory effects in preclinical models by modulating cytokine production and inhibiting NF-κB signaling^66,70^, and was well tolerated in phase I trials at doses up to 900 mg/day^71^. Additionally, both agents exhibit hepatoprotective effects in experimental models, which may support their co-administration with hepatotoxic drugs such as acitretin. Despite acitretin’s established cost-efficacy in moderate to severe psoriasis, its chronic use necessitates regular hepatic monitoring due to its potential hepatotoxicity^72,73^.

Given the hepatotoxic potential of acitretin and the protective roles of melatonin and naringenin, this study aimed to explore the combined and individual effects of these agents in ameliorating acitretin-induced liver injury and to elucidate the underlying protective mechanisms involved. The combination of melatonin and naringenin alongside acitretin offers a promising strategy to enhance therapeutic outcomes while minimizing liver-related adverse effects, potentially improving both efficacy and safety in psoriasis treatment.

## Results

### Melatonin and naringenin co-treatment preserve serum liver function markers

**Fig. 1:**
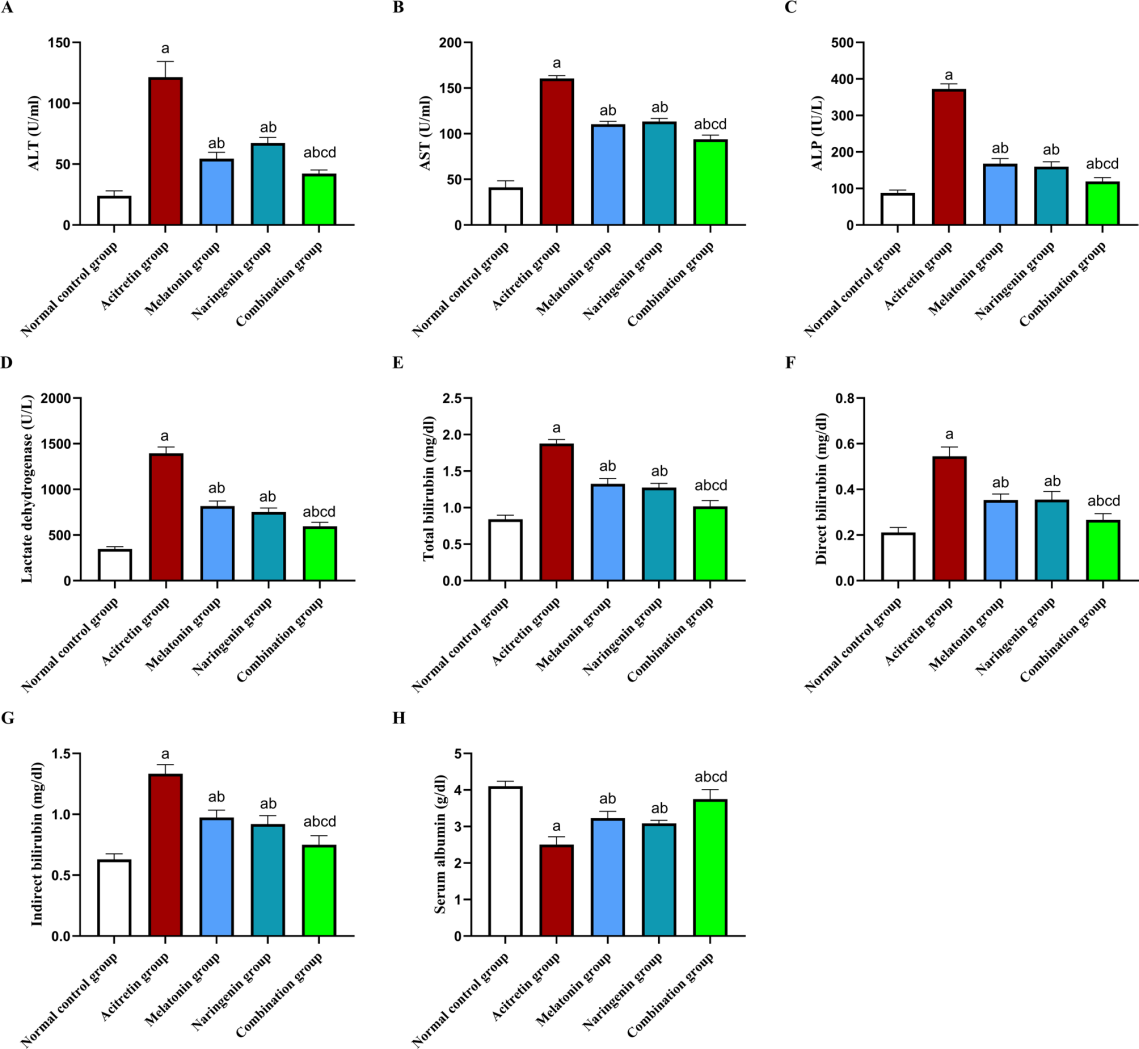
Melatonin and naringenin co-treatment preserve serum liver function markers. (**A**) ALT activity, (**B**) AST activity, (**C**) ALP activity, (**D**) LDH activiety, (**E**) total bilirubin, (**F**) direct bilirubin, (**G**) indirect bilirubin, (**H**) serum albumin. Data (mean ± SD) were subjected to one-way ANOVA, subsequently followed by Tukey’s multiple comparisons. Statistical significance is denoted as follows: ^a^ relative to to the normal control (*P* < 0.05), ^b^ relative to the acitretin group (*P* < 0.05), ^c^ relative to the melatonin group (*P* < 0.05), ^d^ relative to the naringenin group (P < 0.05).

As shown in Fig. 1, the administration of acitretin led to significant increase in the activities of serum enzymes ALT, AST, ALP, and LDH by 407.95%, 289.13%, 325.83%, and 302.33%, respectively, compared to the normal control group. Co-administration of melatonin, naringenin, or their combination significantly decreased these enzyme activities. Specifically, reductions were observed in ALT (55.15%, 44.61%, and 65.25%, respectively), AST (31.18%, 29.22%, and 41.46%, respectively), ALP (55.05%, 57.18%, and 67.93%, respectively), and LDH (41.39%, 46.03%, and 57.25%, respectively) compared to the acitretin group.

In addition, the acitretin group demonstrated elevated levels of total bilirubin, direct bilirubin, and indirect bilirubin by 123.81%, 157.14%, and 111.11%, respectively, compared to the normal control group. The co-administration of melatonin, naringenin, or their combination significantly reduced these levels. Total bilirubin decreased by 29.16%, 32.35%, and 45.67%, respectively; direct bilirubin by 35.76%, 33.92%, and 50.44%, respectively; and indirect bilirubin by 27.21%, 30.96%, and 43.72%, respectively.

Furthermore, the serum albumin levels in the acitretin group were significantly decreased by 38.78% compared to the normal control group. Treatment with melatonin, naringenin, or their combination significantly increased serum albumin levels by 28.81%, 23.23%, and 49.55%, respectively, relative to the acitretin group.

Notably, the combination of melatonin and naringenin exhibited superior effects compared to either treatment alone. This was evident in greater reductions in ALT activity (22.51% and 37.25%), AST activity (14.93% and 17.29%), ALP activity (28.66% and 25.11%), and LDH activity (27.06% and 20.79%) compared to either melatonin or naringenin, respectively. Similarly, significant decreases in total bilirubin (23.31% and 19.69%), direct bilirubin (22.86% and 25%), and indirect bilirubin (22.92% and 18.34%) were observed. Additionally, the combination therapy increased serum albumin levels by 16.10% and 21.36% compared to either melatonin or naringenin, respectively.

## Melatonin and naringenin co-treatment mitigate oxidative damage in hepatic tissue

**Fig. 2:**
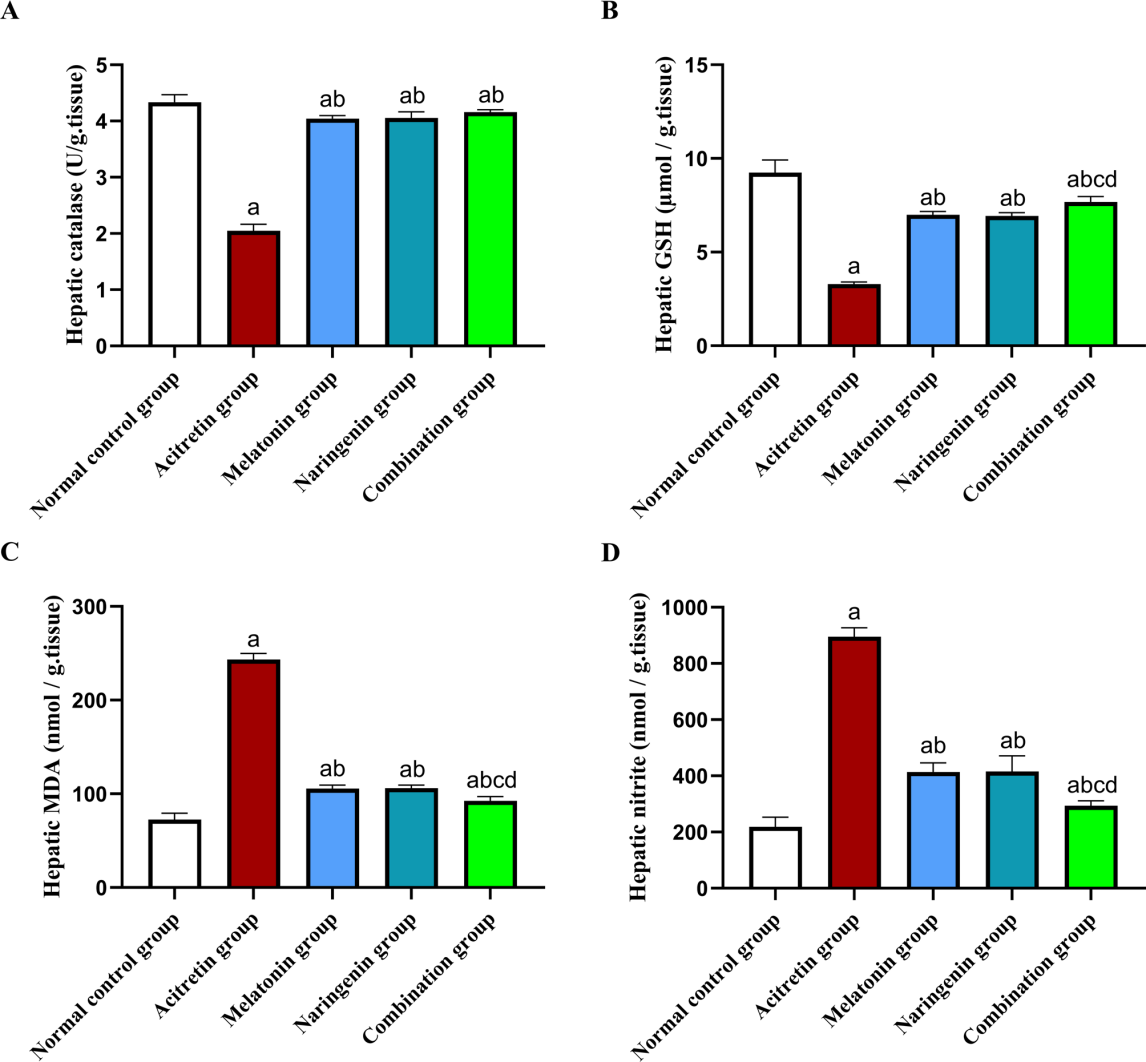
Melatonin and naringenin co-treatment mitigate oxidative damage in hepatic tissue. (**A**) hepatic catalase activety, (**B**) hepatic GSH content, (**C**) hepatic MDA content, (**D**) hepatic nitrite content. GSH: reduced glutation, MDA: malondehyde. Data (mean ± SD) were subjected to one-way ANOVA, subsequently followed by Tukey’s multiple comparisons. Statistical significance is denoted as follows: ^a^ relative to to the normal control (*P* < 0.05), ^b^ relative to the acitretin group (*P* < 0.05), ^c^ relative to the melatonin group (*P* < 0.05), ^d^ relative to the naringenin group (*P* < 0.05).

As shown in Fig. 2, rats in the acitretin group showed a significant reduction in hepatic CAT activity and GSH content by 52.76% and 64.39%, respectively, compared to the normal control group. In contrast, co-administration of melatonin, naringenin, or their combination effectively reversed these reductions, restoring CAT activity by 97.42%, 98.40%, and 103.29%, respectively, and GSH content by 112.57%, 110.45%, and 133.22%, respectively, compared to the acitretin group. Notably, the combination group demonstrated a further significant increase in hepatic GSH levels, surpassing the melatonin and naringenin groups by 9.71% and 10.82%, respectively.

Additionally, hepatic tissues from the acitretin-treated rats showed a significant increase in MDA and nitrite levels, rising by 235.06% and 309%, respectively, compared to the normal control group. Treatment with melatonin, naringenin, or their combination significantly reduced these levels. Specifically, MDA content decreased by 56.57%, 56.37%, and 61.92%, respectively, while nitrite levels decreased by 53.84%, 53.62%, and 67.22%, compared to the acitretin group. Moreover, the combination group showed a further significant reduction in MDA levels by 12.32% and 12.7%, and nitrite levels by 28.99% and 29.33%, compared to either the melatonin or naringenin groups, respectively.

## Melatonin and naringenin co-treatment suppress hepatic pro-inflammatory cytokine levels

**Fig. 3:**
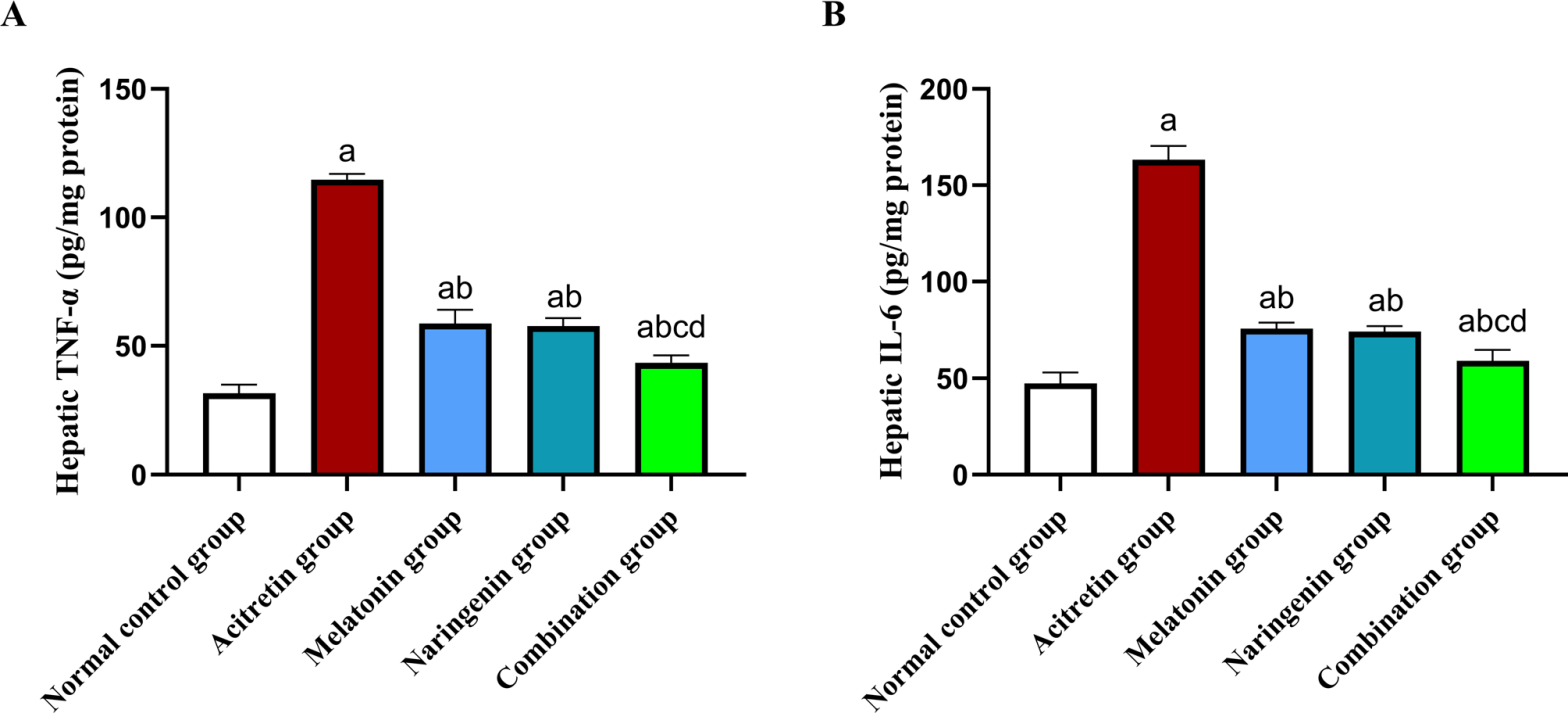
Melatonin and naringenin co-treatment suppress hepatic pro-inflammatory cytokine levels. (**A**) hepatic TNF-α content, (**B**) hepatic IL-6 content. TNF-α: tumor necrosis factor alpha, IL-6: interleukin 6. Data (mean ± SD) were subjected to one-way ANOVA, subsequently followed by Tukey’s multiple comparisons. Statistical significance is denoted as follows: ^a^ relative to to the normal control (*P* < 0.05), ^b^ relative to the acitretin group (*P* < 0.05), ^c^ relative to the melatonin group (*P* < 0.05), ^d^ relative to the naringenin group (*P* < 0.05).

As shown in Fig. 3, hepatic tissues from acitretin-treated rats exhibited a significant increase in TNF-α and IL-6 content, rising by 262.14% and 244.41%, respectively, compared to the normal control group. Treatment with melatonin, naringenin, or their combination significantly mitigated these elevations. Specifically, TNF-α levels were reduced by 48.83%, 49.74%, and 62.14%, respectively, while IL-6 levels decreased by 53.73%, 54.6%, and 63.92%, respectively relative to the acitretin group. Furthermore, the combination group demonstrated an additional significant reduction in TNF-α levels by 26.01% and 24.67%, and in IL-6 levels by 22.04% and 20.53%, respectively compared to the melatonin and naringenin groups individually.

## Melatonin and naringenin co-treatment improve hepatic histopathological features

**Fig. 4:**
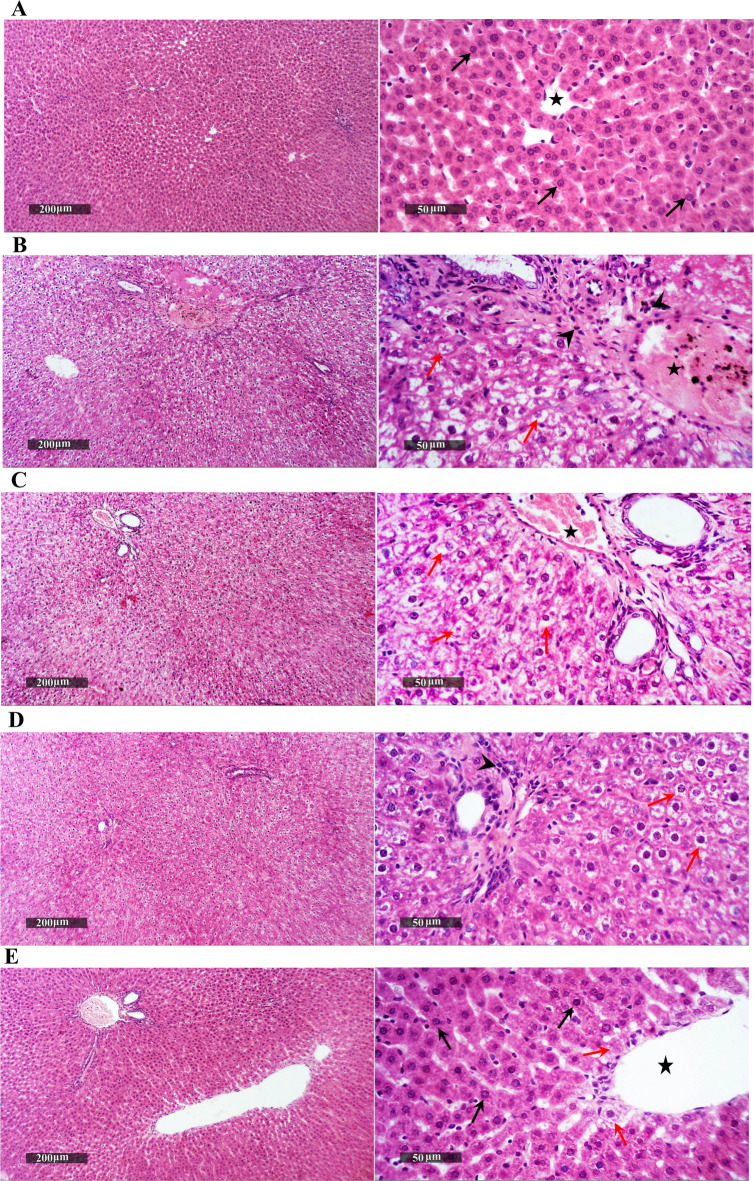
Melatonin and naringenin co-treatment improve hepatic histopathological features. (**A**) Normal control group showing well-organized hepatic parenchyma with vesicular intact hepatocytes and discernible nuclei (black arrow), intact hepatic vasculature (star), and normal sinusoids free from inflammatory infiltration. (**B**) Acitretin group displaying extensive pan-lobular hepatocellular vacuolar degeneration, multiple focal necrotic changes (red arrow), moderate periportal inflammatory cell infiltration (arrowhead), and moderate dilatation of hepatic vasculature (star). (**C**) Melatonin group showing slight improvement with persistent degenerative changes and minimal abnormal inflammatory cell infiltrates (arrowhead). (**D**) Naringenin treated group exhibiting moderate hepatocellular degeneration and necrosis (red arrow), occasional abnormal inflammatory infiltrates (arrowhead), and intact vasculature. (**E**) Combination group demonstrating marked hepatoprotective effect with abundant intact hepatocytes (black arrow), minimal degenerative changes (red arrow), mild occasional inflammatory infiltration (arrowhead), and moderately dilated vasculature (star).

As shown in Fig. 4, histological examination of liver sections stained with H&E revealed distinct morphological changes across the experimental groups. The control group exhibited well-preserved hepatic architecture, characterized by orderly arranged hepatic cords, vesicular intact hepatocytes with clear nuclei, and normal vasculature without any signs of inflammation or cellular infiltration. In contrast, liver sections from the acitretin-treated group displayed marked histopathological alterations, including widespread hepatocellular vacuolar degeneration and multiple foci of necrosis. These changes were accompanied by moderate periportal inflammatory cell infiltration and dilatation of hepatic blood vessels, indicating significant hepatic injury. Liver sections from the melatonin-treated group displayed a modest improvement in hepatic architecture. While degenerative changes were still observed, the extent of inflammation appeared reduced, with only minimal records of abnormal inflammatory cell infiltration compared to the acitretin group. Naringenin-treated samples showed moderate restoration of hepatic morphology, with persistent but less extensive degenerative and necrotic changes. Occasional mild inflammatory infiltration was observed, while vascular structures appeared relatively intact. The most notable improvement was observed in the combination treatment group, which showed clear preservation of liver architecture, with widespread intact hepatocytes throughout the lobules and only minimal evidence of degenerative alterations. Mild, occasional inflammatory infiltration was still observed, accompanied by moderate vascular dilatation.

## Melatonin and naringenin co-treatment downregulate HMGB1/TLR4/NF-κB pathway

**Fig. 5:**
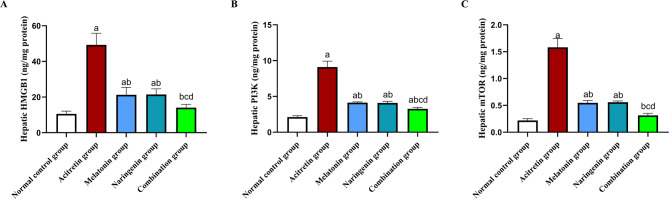
Melatonin and naringenin co-treatment downregulate hepatic content of HMGB1(**A**), PI3K (**B**), and mTOR (**C**). HMGB1: high mobility group box 1, PI3K: phosphoinositide 3-kinases, mTOR: mammalian target of rapamycin. Data (mean ± SD) were subjected to one-way ANOVA, subsequently followed by Tukey’s multiple comparisons. Statistical significance is denoted as follows: ^a^ relative to to the normal control (*P* < 0.05), ^b^ relative to the acitretin group (*P* < 0.05), ^c^ relative to the melatonin group (*P* < 0.05), ^d^ relative to the naringenin group (*P* < 0.05).

As illustrated in Fig. 5, ELISA measurements revealed a significant increase in hepatic content of HMGB1, PI3K, and mTOR in the acitretin-treated group, with elevations of 367.01%, 329.72%, and 618.18%, respectively, compared to the normal control group. Co-administration of melatonin, naringenin, or their combination significantly reduced these levels. Specifically, HMGB1 levels were decreased by 56.81%, 56.38%, and 71.44%, respectively; PI3K levels by 54.66%, 55.10%, and 64.1%; and mTOR levels by 65.22%, 64.59%, and 80.4%, compared to the acitretin group. Notably, the combination treatment resulted in even greater reductions in hepatic HMGB1 (33.88% and 34.53%), PI3K (20.82% and 20.05%), and mTOR (43.64% and 44.64%) levels compared to melatonin or naringenin, respectively.

**Fig. 6:**
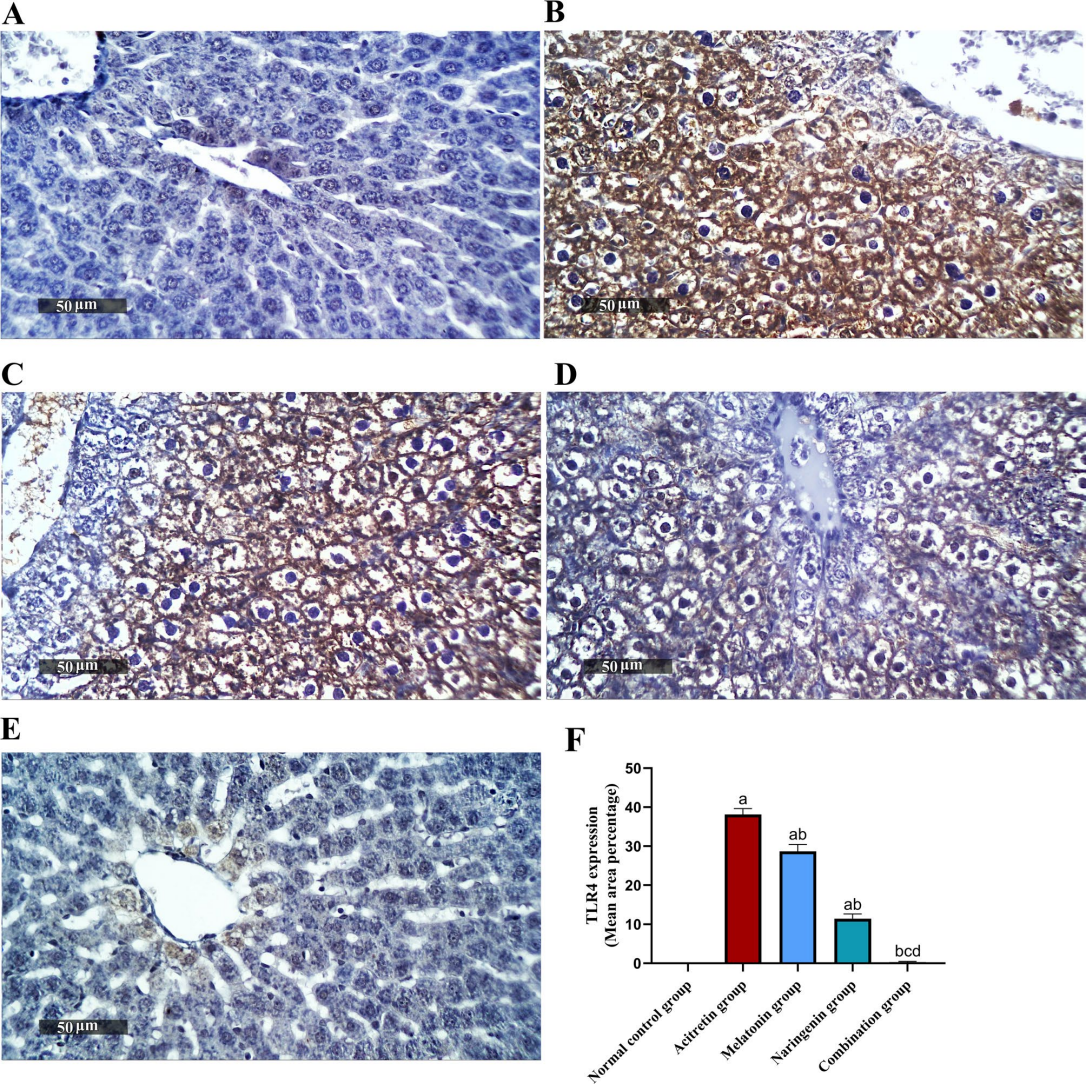
Melatonin and naringenin co-treatment downregulate hepatic expression of TLR4. (**A**) Normal control group, (**B**) Acitretin group, (**C**) Melatonin group, (**D**) Naringenin group (**E**) Combination group, (**F**) Area percentage of hepatocellular expression. Data (mean ± SD) were subjected to one-way ANOVA, subsequently followed by Tukey’s multiple comparisons. Statistical significance is denoted as follows: ^a^ relative to to the normal control (*P* < 0.05), ^b^ relative to the acitretin group (*P* < 0.05), ^c^ relative to the melatonin group (*P* < 0.05), ^d^ relative to the naringenin group (*P* < 0.05).

**Fig. 7:**
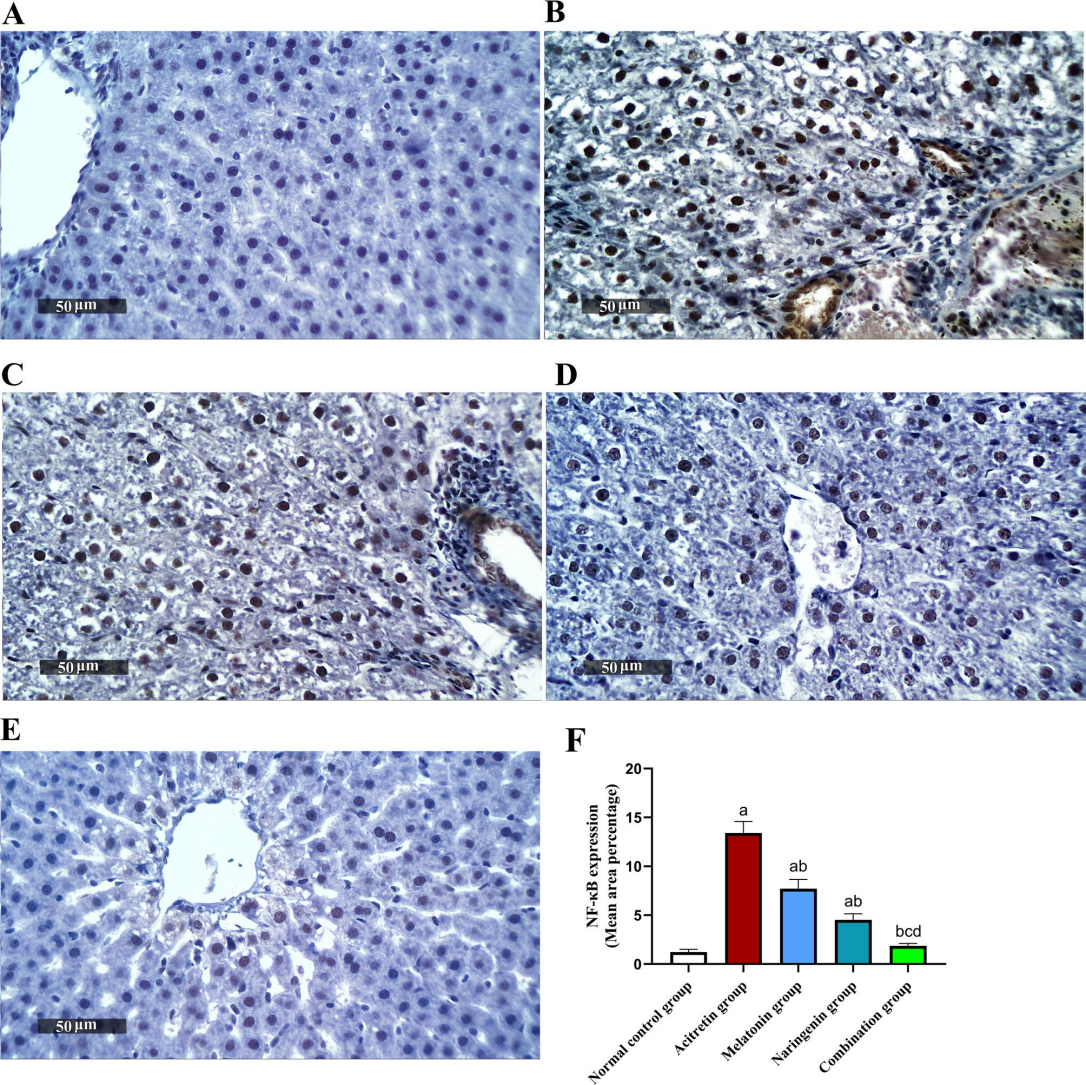
Melatonin and naringenin co-treatment downregulate hepatic expression of NF-κB. (**A**) Normal control group, (**B**) Acitretin group, (**C**) Melatonin group, (**D**) Naringenin group (**E**) Combination group, (**F**) Area percentage of hepatocellular expression. Data (mean ± SD) were subjected to one-way ANOVA, subsequently followed by Tukey’s multiple comparisons. Statistical significance is denoted as follows: ^a^ relative to to the normal control (*P* < 0.05), ^b^ relative to the acitretin group (*P* < 0.05), ^c^ relative to the melatonin group (*P* < 0.05), ^d^ relative to the naringenin group (*P* < 0.05).

As shown in Figs. 6 and 7, immunohistochemical analysis revealed a significant increase in the expression of TLR4 and NF-κB in the acitretin-treated group, by 47,587.5% and 998.36%, respectively, compared to the normal control group. Treatment with melatonin led to a reduction in TLR4 and NF-κB expression by 24.82% and 42.39%, respectively, while naringenin treatment resulted in reductions of 70.07% and 66.27%, respectively, relative to the acitretin group. The combination treatment demonstrated the most pronounced effect, decreasing TLR4 expression by99.21% relative to the acitretin group and by 98.95% and 97.37% compared to melatonin and naringenin monotherapy, respectively, with levels comparable to those in the control group. Similarly, NF-κB expression in the combination group was reduced by 86.19% compared to the acitretin group, and by 76.04% and 59.07% relative to melatonin and naringenin alone, respectively, again showing no significant difference from the normal control.

## Melatonin and naringenin co-treatment suppress hepatic caspase-3 expression

**Fig. 8:**
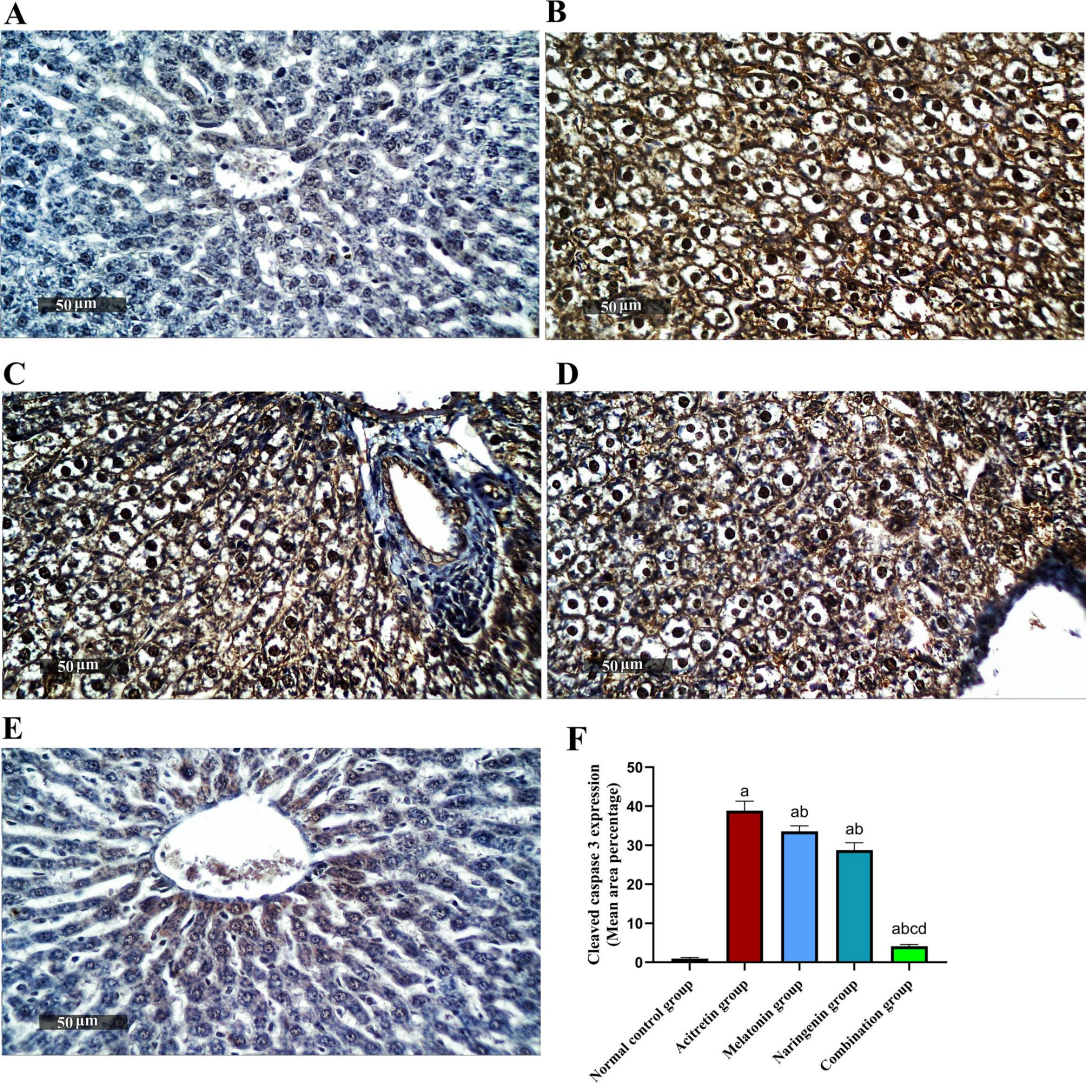
Melatonin and naringenin co-treatment suppress hepatic expression of caspase-3. (**A**) Normal control group, (**B**) Acitretin group, (**C**) Melatonin group, (**D**) Naringenin group (**E**) Combination group, (**F**) Area percentage of hepatocellular expression. Data (mean ± SD) were subjected to one-way ANOVA, subsequently followed by Tukey’s multiple comparisons. Statistical significance is denoted as follows: ^a^ relative to to the normal control (*P* < 0.05), ^b^ relative to the acitretin group (*P* < 0.05), ^c^ relative to the melatonin group (*P* < 0.05), ^d^ relative to the naringenin group (*P* < 0.05).

As shown in Fig. 8, immunohistochemical analysis for cleaved caspase-3 revealed a significant increase in its expression in the acitretin-treated group, with a 3991.58% elevation compared to the normal control. Treatment with melatonin and naringenin reduced caspase-3 expression by 13.68% and 25.98%, respectively, relative to the acitretin group. Notably, the combination therapy produced a significantly greater reduction of 89.45% compared to the acitretin group, and by 87.78% and 85.75% relative to melatonin and naringenin, respectively.

## Melatonin and naringenin co-treatment attenuate hepatic fibrogenic markers (TGF-β and MMP-9)

**Fig. 9:**
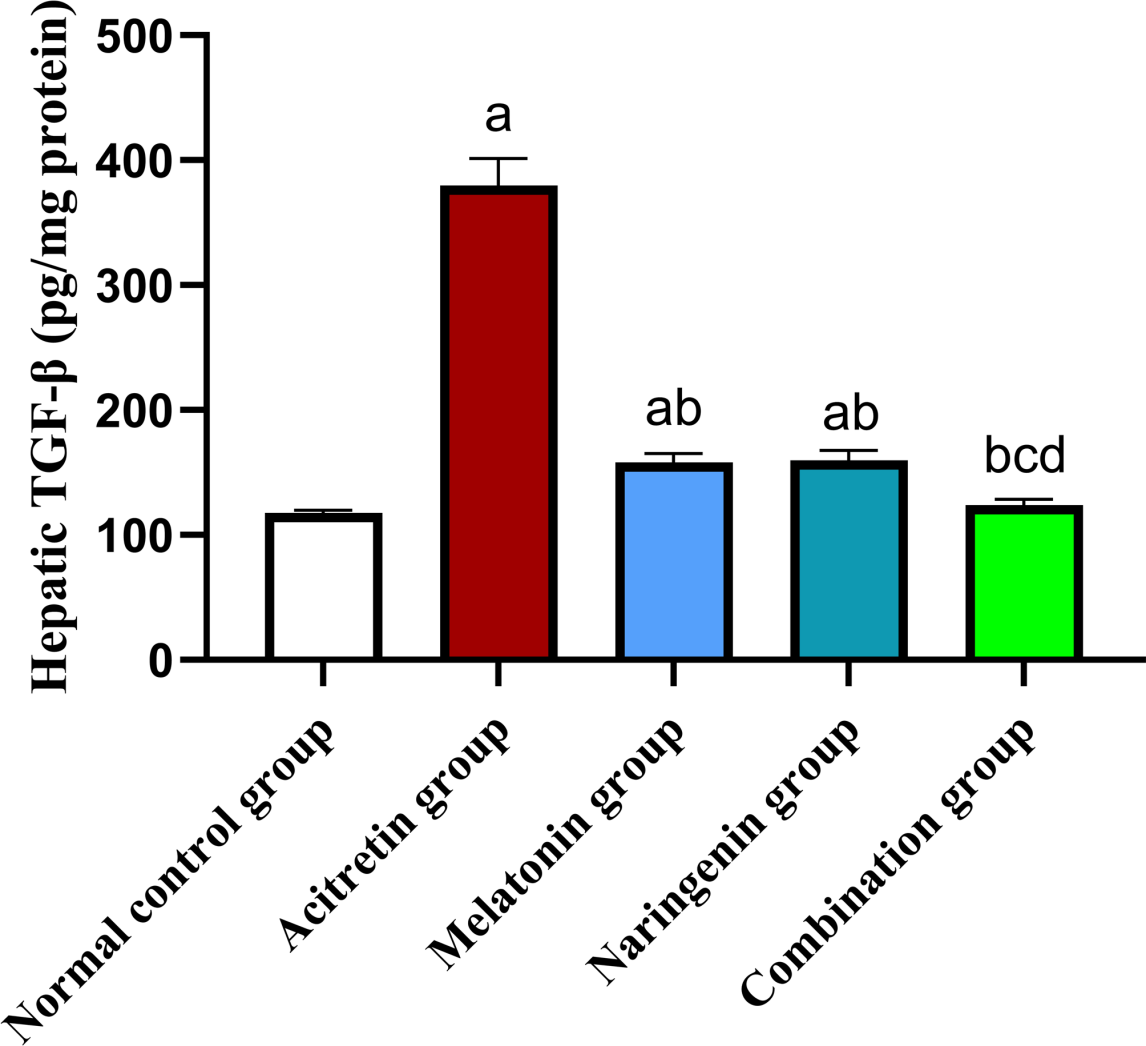
Melatonin and naringenin co-treatment downregulate hepatic content of TGF-β. TGF-β: transforming growth factor beta. Data (mean ± SD) were subjected to one-way ANOVA, subsequently followed by Tukey’s multiple comparisons. Statistical significance is denoted as follows: ^a^ relative to to the normal control (*P* < 0.05), ^b^relative to the acitretin group (*P* < 0.05), ^c^ relative to the melatonin group (*P* < 0.05),^d^ relative to the naringenin group (*P* < 0.05).

Hepatic tissues from the acitretin-treated group exhibited a significant increase in TGF-β content by 222.2% compared to the normal control group. Co-administration of melatonin, naringenin, or their combination significantly reduced hepatic TGF-β levels by 58.34%, 57.97%, and 67.38%, respectively, compared to the acitretin group. Furthermore, the combination of melatonin and naringenin achieved a greater reduction in TGF-β levels by 21.69% and 22.38% compared to melatonin or naringenin, respectively (Fig. 9).

**Fig. 10:**
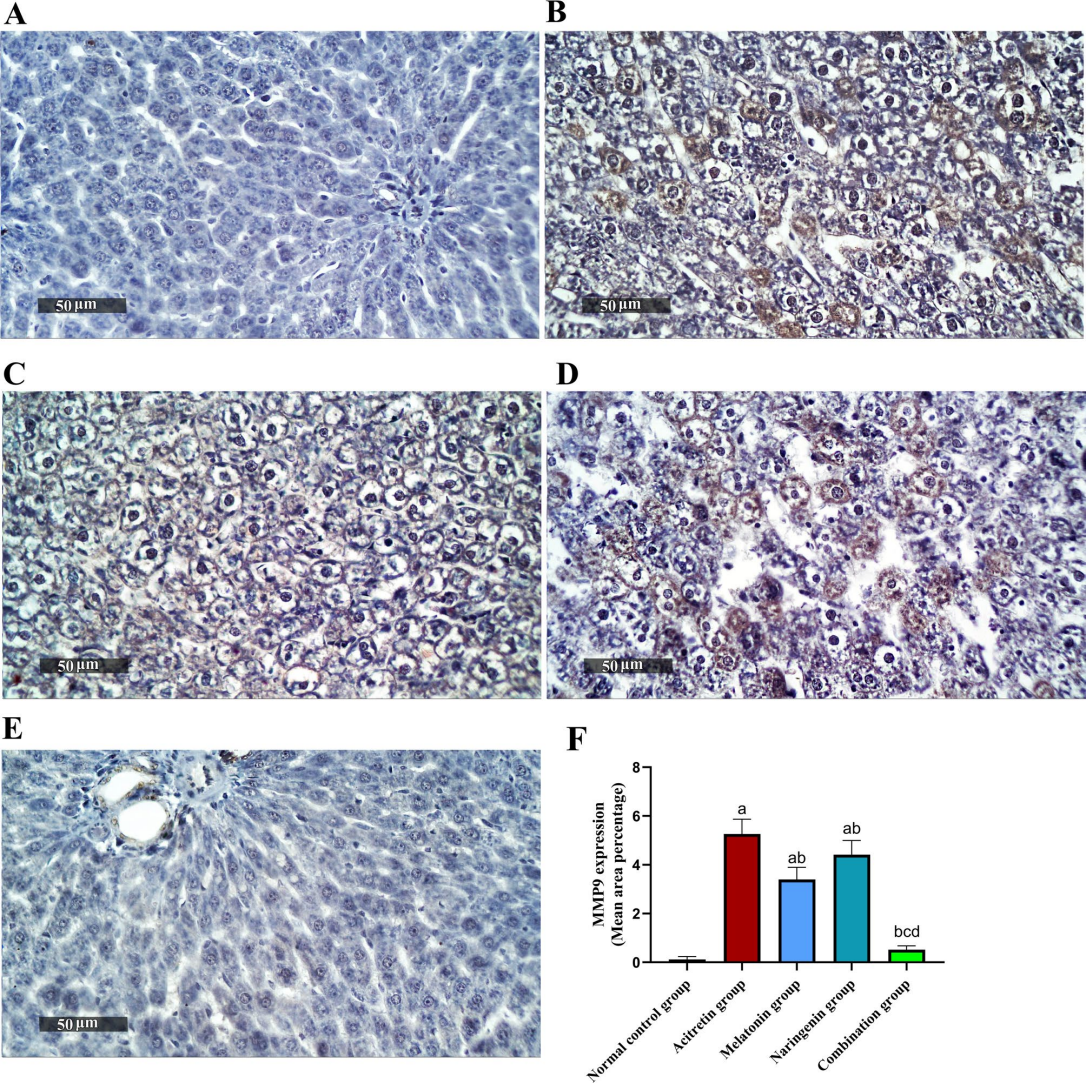
Melatonin and naringenin co-treatment downregulate hepatic expression of MMP9. (**A**) Normal control group, (**B**) Acitretin group, (**C**) Melatonin group, (**D**) Naringenin group (**E**) Combination group, (**F**) Area percentage of hepatocellular expression. Data (mean ± SD) were subjected to one-way ANOVA, subsequently followed by Tukey’s multiple comparisons. Statistical significance is denoted as follows:^a^ relative to to the normal control (P < 0.05),^b^ relative to the acitretin group (*P* < 0.05),^c^ relative to the melatonin group (*P* < 0.05),^d^ relative to the naringenin group (P < 0.05).

As shown in Fig. 10, immunohistochemical analysis of MMP-9 revealed a significant increase in expression in the acitretin-treated group, with a 4291.67% elevation compared to the normal control. Treatment with melatonin and naringenin reduced MMP-9 expression by 35.44% and 16.08%, respectively, relative to the acitretin group. Notably, the combination treatment produced a more significant reduction of 90.13% compared to the acitretin group, and by 84.71% and 88.24% compared to melatonin and naringenin monotherapy, respectively. MMP-9 expression in the combination group showed no significant difference from the control group.

## Melatonin and naringenin co-treatment modulate *JAK2* and *STAT3* pathway

**Fig. 11:**
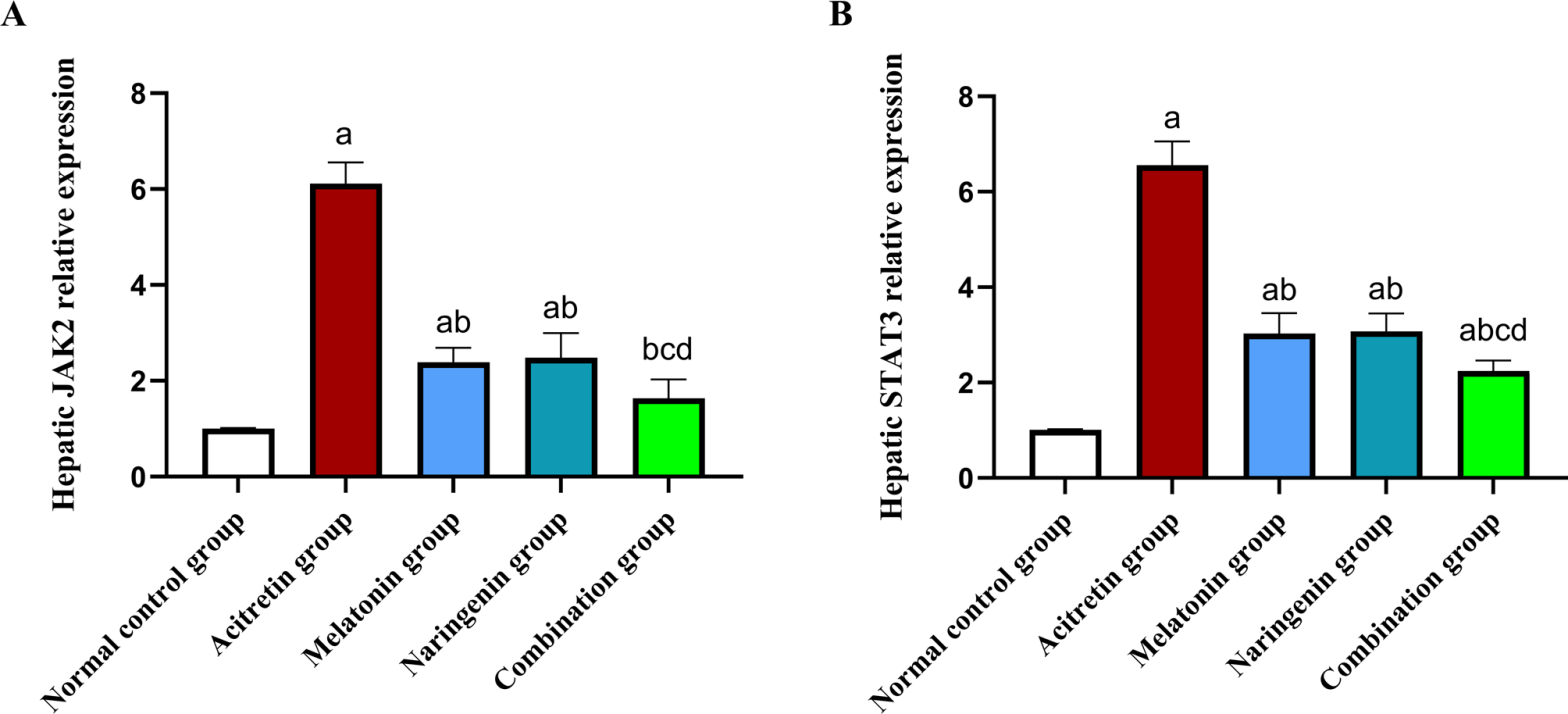
Melatonin and naringenin co-treatment modulate *JAK2* (**A**) and *STAT3* (**B**) gene expression. *JAK2*: janus kinase 2, *STAT3*: signal transducer and activator of transcription 3. Data (mean ± SD) were subjected to one-way ANOVA, subsequently followed by Tukey’s multiple comparisons. Statistical significance is denoted as follows: ^a^ relative to the normal control (*P* < 0.05), ^b^ relative to the acitretin group (*P* < 0.05), ^c^ relative to the melatonin group (*P* < 0.05),^d^ relative to the naringenin group (*P* < 0.05).

As shown in Fig. 11, rats treated with acitretin exhibited a significant increase in the gene expression of *JAK2* and *STAT3*, rising by 512% and 549.5%, respectively, compared to the normal control group. Co-administration of melatonin, naringenin, or their combination significantly reduced the expression levels of these genes. Specifically, *JAK2* expression decreased by 60.93%, 59.46%, and 73.35%, respectively, while *STAT3* expression was reduced by 53.79%, 53.02%, and 65.84%, relative to the acitretin group. Notably, the combination treatment achieved an even greater reduction in *JAK2* expression (31.8% and 34.27%) and *STAT3* expression (26.07% and 27.27%) compared to melatonin or naringenin, respectively.

## Discussion

In the field of pharmaceutical practice, DILI is a leading cause of drug withdrawal, usage restrictions, and project termination^5^. Despite its effectiveness in treating moderate to severe conditions of psoriasis and its cost-efficiency, acitretin has been linked to hepatotoxic effects. These include transient elevations in liver enzymes and, in rare instances, severe hepatotoxicity^14,16,19,72,74–76^. However, the exact mechanism underlying its hepatotoxicity remains unclear. Available data suggest that its liver-damaging effects are associated with elevated liver enzymes, increased LDH levels, mitochondrial dysfunction leading to apoptosis and necrosis, as well as cholestatic injury^13,14,18^. Additionally, effective mitigation strategies for these adverse effects are still lacking.

This study aimed to investigate the protective effects of melatonin and naringenin, either individually or in combination, against acitretin-induced liver injury. The results revealed that acitretin administration significantly elevated serum liver enzyme levels, increased hepatic oxidative stress markers and triggered inflammatory responses. However, treatment with melatonin and naringenin effectively mitigated these adverse effects, with their combined use demonstrating superior hepatoprotective benefits.

The biochemical analysis revealed significant elevation in serum ALT, AST, ALP, and LDH levels in the acitretin-treated group, indicating hepatic dysfunction. ALT and AST are well-established markers of hepatocellular injury^22,25,77–79^, while ALP and LDH are indicators of cholestasis and cellular damage, respectively^77,80,81^. This finding aligns with previous studies demonstrating acitretin-induced hepatotoxicity characterized by disrupted liver enzyme homeostasis^14,16,18^. The co-administration of melatonin and naringenin significantly reduced these elevated enzymes, restoring hepatic function. The observed hepatoprotective effect can be attributed to the well-documented antioxidant, anti-inflammatory, and anti-apoptotic properties of both compounds^40–42,44–47,49,53,55,56,58–60,82^. These findings are further supported by the observed increase in serum bilirubin levels (direct, indirect, and total) and the reduction in serum albumin following acitretin administration. The coadministration of melatonin, naringenin, and their combination effectively mitigated these alterations, reinforcing their hepatoprotective potential. Notably, the combination treatment demonstrated a more pronounced reduction in these biomarkers, suggesting a potential additive or synergistic hepatoprotective mechanism.

Oxidative stress is a key contributor to DILI^21,83^. Acitretin has been previously documented to induce mitochondrial dysfunction^13^, which can trigger the overproduction of reactive oxygen species and subsequent oxidative stress^84,85^. The present findings support this mechanism, as indicated by increased hepatic MDA and nitrite levels alongside diminished antioxidant enzyme activity. MDA, a byproduct of lipid peroxidation, serves as an indicator of oxidative damage to cellular membranes^86^, whereas elevated nitrite levels suggest enhanced nitric oxide production, which exacerbates oxidative stress and inflammation^87,88^. The observed reduction in catalase and GSH activities further highlights the impairment of the liver’s antioxidant defense system. Notably, treatment with melatonin and naringenin effectively counteracted these oxidative stress markers by restoring catalase and GSH activities. Their potent antioxidant properties likely underline this protective effect, as both compounds have been reported to strengthen cellular antioxidant defenses^40,89–92^. Among the treatment groups, the combination treatment demonstrated the greatest efficacy in alleviating oxidative damage, reinforcing the concept of a complementary mechanism between these agents.

These findings were further consolidated by the histopathological observations, which revealed significant hepatic damage in the acitretin-treated group, including hepatocyte necrosis, inflammatory cell infiltration, and vacuolar degeneration indicative of severe cytotoxic and inflammatory effects. However, treatment with naringenin and melatonin effectively ameliorated these pathological changes, preserving hepatic architecture and attenuating inflammation.

Heightened oxidative stress accelerates cellular damage, leading to the release of DAMPs like HMGB1, which activate inflammatory pathways via TLR4^20,21^. This activation triggers the MyD88-dependent pathway, stimulating the PI3K/Akt/mTOR cascade^22–25^. Consequently, NF-κB is activated, promoting the release of pro-inflammatory cytokines such as TNF-α, IL-1β, IL-6, IL-12, and COX-2^26,27^.

In the present study, acitretin administration significantly upregulated the expression of HMGB1, TLR4, PI3K, mTOR, and NF-κB, along with increased hepatic levels of TNF-α and IL-6, indicating the activation of inflammatory and pro-apoptotic signaling pathways. These findings suggest that acitretin-induced hepatotoxicity is mediated, at least in part, through the HMGB1/TLR4/MyD88 axis, leading to downstream activation of PI3K/Akt/mTOR and NF-κB, which subsequently amplifies the inflammatory response. Notably, treatment with melatonin and naringenin effectively mitigated these effects by downregulating the expression of TLR4 and its associated signaling components while reducing TNF-α and IL-6 levels. The combination therapy demonstrated the most pronounced suppression of these inflammatory mediators, suggesting a complementary anti-inflammatory effect that may highlight its superior hepatoprotective potential.

Activation of NF-κB is closely associated with the upregulation of MMP-9, a key mediator of inflammation and tissue remodeling, as evidenced in various pathological conditions^28,29^. In this study, the observed increase in NF-κB activation following acitretin administration was accompanied by a significant overexpression of hepatic MMP-9, indicating heightened inflammation and tissue remodeling. This suggests that acitretin-induced liver injury involves NF-κB-mediated M1 macrophage activation, leading to extracellular matrix degradation and hepatic damage^93,94^. Several studies in the literature support the existence of a functional NF-κB/MMP-9 signaling axis in various models of liver injury and inflammation. NF-κB activation enhances MMP-9 transcription by promoting p65 nuclear translocation and DNA binding to MMP-9 promoter regions, leading to extracellular matrix degradation and inflammatory cell recruitment^95–100^. In hepatic and vascular tissues, suppression of NF-κB activity is consistently associated with reduced MMP-9 expression and inflammation^97,98,100^.

Treatment with melatonin and naringenin effectively suppressed NF-κB activation and reduced MMP-9 expression, demonstrating their anti-inflammatory and hepatoprotective properties. Notably, the combination therapy exhibited the strongest inhibitory effect, reinforcing its potential complementary mechanism in mitigating acitretin-induced inflammatory damage. Importantly, melatonin has been shown not only to inhibit NF-κB activation and MMP-9 transcription^98,100,101^, but also, to directly bind to the catalytic domain of MMP-9, thereby suppressing its enzymatic activity independent of transcriptional regulation^102^.

The inflammatory response mediated by TNF-α plays a crucial role in hepatocyte apoptosis through the activation of caspase-3^30,31^. Meanwhile, IL-6, secreted by Kupffer cells or hepatocytes, activates the JAK/STAT3 signaling pathway, which is a key regulator of inflammation and stress responses, including various forms of DILI^32,103–105^. Dysregulation of this pathway can contribute to chronic inflammation, fibrosis, immune deficiencies, and even malignancies^33,106^. In the present study, acitretin administration significantly increased hepatic caspase-3 expression, confirming its role in apoptosis-mediated hepatocyte damage. This aligns with the observed elevation in TNF-α, reinforcing the link between inflammatory signaling and hepatocellular apoptosis. Additionally, acitretin-treated rats exhibited a marked upregulation of JAK and STAT3 gene expression, indicating activation of this pro-inflammatory pathway, which has been implicated in various cases of DILI^103,104,106^. Treatment with melatonin and naringenin effectively suppressed caspase-3 immunostaining and downregulated *JAK*/*STAT3* expression, demonstrating their anti-apoptotic and anti-inflammatory properties. Notably, the combination group exhibited the most substantial reduction in these markers, confirming its superiority in protecting against acitretin-induced liver injury.

In chronic liver disease, TGF-β plays a pivotal role in disease progression by activating hepatic stellate cells, leading to fibrosis, cirrhosis, and eventually hepatocellular carcinoma^34^. Elevated TGF-β levels are a hallmark of chronic liver damage, driving the transformation of stellate cells into fibrogenic myofibroblasts and promoting hepatocyte apoptosis, ultimately contributing to liver fibrosis^35,36^. Myofibroblasts further exacerbate fibrosis by producing excessive collagen, while MMP-9 facilitates extracellular matrix degradation, worsening hepatic injury^37,38^. In the present study, acitretin administration significantly elevated hepatic TGF-β levels and MMP-9 expression, suggesting its role in fibrogenic activation and extracellular matrix remodeling. These findings indicate that acitretin-induced liver injury may contribute to fibrosis through TGF-β-mediated stellate cell activation and MMP-9-driven tissue remodeling. Treatment with melatonin and naringenin effectively suppressed these fibrogenic markers, demonstrating their antifibrotic potential. Notably, the combination therapy showed the most substantial reduction in TGF-β and MMP-9 levels, suggesting a complementary protective mechanism against acitretin-induced hepatic fibrosis.

## Conclusion

**Fig. 12:**
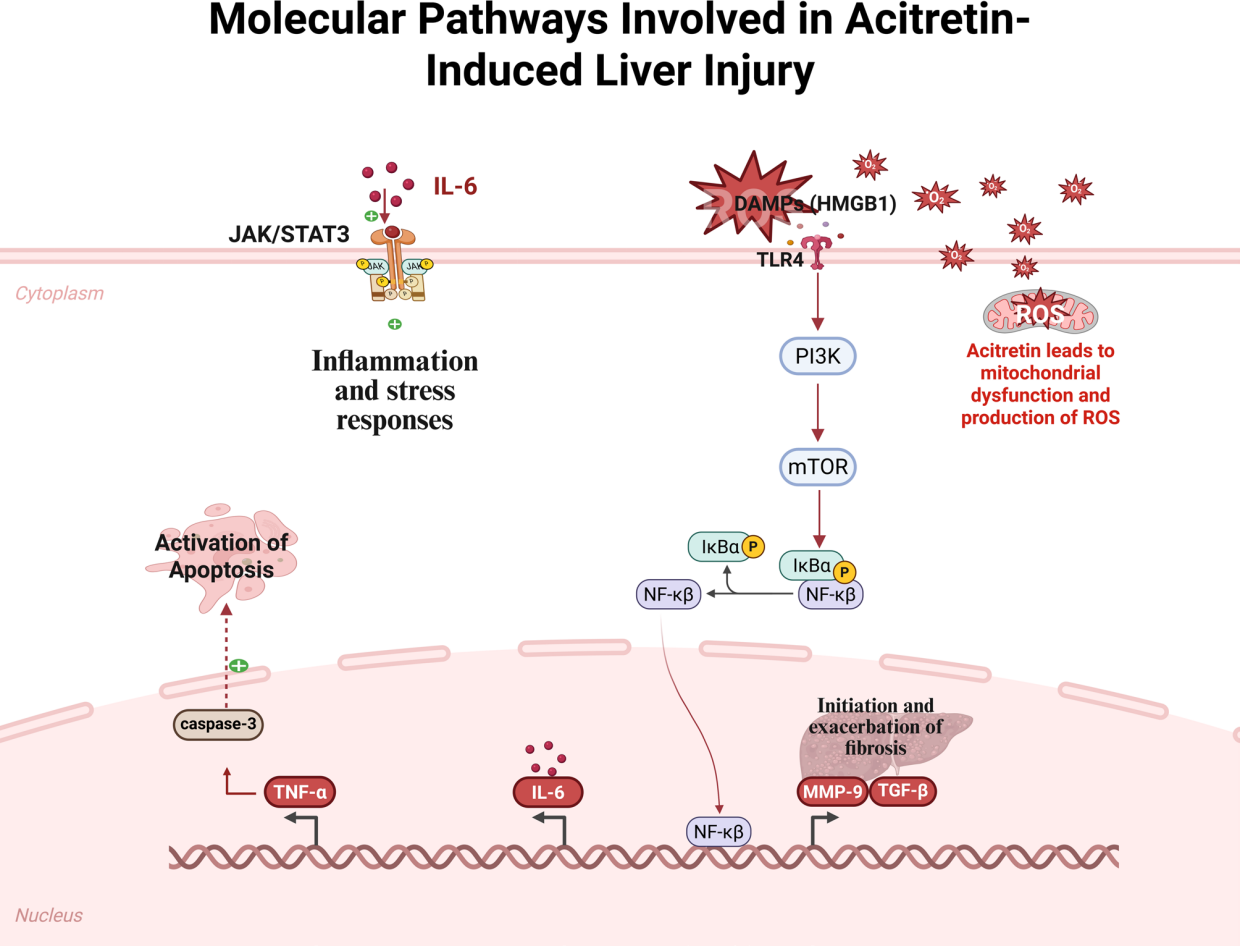
Molecular pathways involved in acitretin-induced hepatotoxicity. Acitretin induces mitochondrial dysfunction and excessive generation of reactive oxygen species (ROS), resulting in oxidative stress and the release of damage-associated molecular patterns (DAMPs), notably high-mobility group box 1 (HMGB1). HMGB1 activates the Toll-like receptor 4 (TLR4), triggering the phosphoinositide 3-kinase (PI3K)/mammalian target of rapamycin (mTOR) signaling cascade, which leads to the nuclear translocation of nuclear factor kappa B (NF-κB). This results in the upregulation of pro-inflammatory cytokines, including tumor necrosis factor-alpha (TNF-α) and interleukin-6 (IL-6), as well as matrix remodeling proteins such as matrix metalloproteinase-9 (MMP-9) and transforming growth factor-beta (TGF-β). Additionally, IL-6 plays a key role in activating the Janus kinase/signal transducer and activator of transcription 3 (JAK/STAT3) pathway, further amplifying inflammatory and fibrotic responses. Apoptotic signaling is also promoted via caspase-3 activation, contributing to acitretin-induced liver injury. Created in BioRender. Elgindy, A. (2025) https://BioRender.com/rzudedw.

This study demonstrates that acitretin induces significant hepatotoxicity, as evidenced by elevated liver enzyme levels, oxidative stress, inflammatory cytokine production, and apoptotic and fibrotic markers. These changes are mediated through activation of the HMGB1/TLR4/NF-κB signaling pathway, the JAK/STAT3 cascade, and TGF-β/MMP-9-driven fibrosis (Fig. 12). Treatment with melatonin and naringenin effectively mitigated these effects by restoring hepatic antioxidant defenses, suppressing inflammatory signaling, and reducing apoptosis and fibrosis. Notably, the combination treatment exhibited superior hepatoprotective effects compared to each drug alone, suggesting a potential complementary mechanism. These findings highlight the therapeutic potential of melatonin and naringenin as promising adjuncts to acitretin therapy, offering a safer and more effective approach for managing psoriasis while minimizing liver-related complications. Further studies are warranted to explore their clinical applicability and underlying interactions. In addition, future mechanistic investigations and binding activity studies are needed to clarify the molecular interactions involved.

## Methods

### Animals

Fifty male Sprague Dawley rats (150–170 g, 8–10 weeks old) were obtained from Zel Al-Nakheel for Supplying Laboratory Animals and Feed Company in Giza, Egypt. The animals were housed for one week in the animal facility at the Faculty of Pharmacy, Tanta University, allowing for acclimatization before the experiment. Rats were kept in a regulated environment with a temperature of 22 ± 2 °C, humidity levels of 50–60%, and a 12-h light/dark cycle. The rats were provided with unrestricted access to standard laboratory food and water during the study.

The experimental protocol was approved by the Research Ethics Committee of the Faculty of Pharmacy, Tanta University (REC-TP), adhering to the guidelines of the Council for International Organization of Medical Sciences (CIOMS) and local regulations (approval code: TP/RE/5/24 Ph-5). Euthanasia was performed in accordance with the American Veterinary Medical Association (AVMA) Guidelines for the Euthanasia of Animals (2020). The study is reported in accordance with the ARRIVE guidelines (PLoS Biol 8(6), e1000412, 2010).

## Chemicals

Acitretin (Unitrin®, 25 mg) was supplied by Unipharma (Cairo, Egypt). Melatonin was acquired from Solarbio (Beijing, China), and naringenin was obtained from Axenic Lab (Oak Park, Australia). All compounds were suspended in freshly prepared 0.5% carboxymethyl cellulose (CMC) immediately prior to administration via oral gavage.

## Experimental design

Fifty rats were randomly divided into five groups, each consisting of ten rats. The **normal control group** received 0.5% CMC (4 ml/kg, P.O.) daily for four weeks. In the **acitretin group**, rats were administered acitretin (60 mg/kg, P.O.) on alternate days for the same duration. The **melatonin group** received both acitretin (60 mg/kg, P.O.) every other day and melatonin (10 mg/kg, P.O.) daily for four weeks, with melatonin given one hour before acitretin. Similarly, the **naringenin group** was treated with acitretin (60 mg/kg, P.O.) on alternate days alongside daily naringenin (20 mg/kg, P.O.) for four weeks, administered an hour prior to acitretin. Lastly, the **combination group** was given acitretin (60 mg/kg, P.O.) every other day, in addition to daily doses of melatonin (10 mg/kg, P.O.) and naringenin (20 mg/kg, P.O.) for four weeks.

At the conclusion of the experimental protocol, rats were euthanized with pentobarbital sodium (100 mg/kg, intraperitoneally), and blood samples were collected via retro-orbital sinus exsanguination. The collected blood samples were centrifuged to separate serum, which was then stored at −20 ℃ for subsequent hepatic function tests. The animals were then sacrificed by cervical dislocation. Liver tissues were collected and divided into two portions: one of the two parts was fixed in 10% neutral-buffered formalin for histopathological and immunohistochemical examination, while the other part was utilized for biochemical analyses, including oxidative stress marker determination, enzyme-linked immunosorbent assays (ELISA), and real-time quantitative reverse transcription polymerase chain reaction (qRT-PCR) analyses.

## Explanation of utilized doses

The dosage of acitretin was established following a preliminary study conducted in rats, which involved the administration of four different doses: 10, 30, 60, and 100 mg/kg every other day for a duration of four weeks. Full details of this preliminary study are provided in the “Supplementary Data” file. The summarized outcomes are presented in Table S1, and corresponding graphical and histological findings are shown in Figures S1 and S2, respectively. Among these doses, the 60 mg/kg dosage exhibited the most notable and statistically significant effects. The selection of these doses was based on the consideration that the maximal human therapeutic dose is 50 mg daily^107^. This equivalent dosage in rats would amount to 5.2 mg/kg daily for rats or 10.4 mg/kg every other day, taking into account the long elimination half-life of acitretin (49 h)^10,108^. Higher dosage ranges were employed in the preliminary study to enable the clear identification of the mechanistic pathways underlying its toxic effects in a reproducible manner. The selected dose was far below the reported oral LD_50_ of acitretin in rats (> 4000 mg/kg) ^109^, ensuring safety while allowing reliable hepatotoxic induction. Moreover, since previous studies have not examined acitretin-induced hepatotoxicity in vivo, conducting a preliminary study was essential to ensure consistent outcomes.

In the literature, the oral administration of melatonin has been investigated across a dosage range of 5–15 mg/kg/day^41,110–114^. Among these doses, the dose of 10 mg/kg/day has been approved as the optimal dosage for effectively reducing high-carbohydrate high-fat (HCHF) diet-induced non-alcoholic fatty liver disease (NAFLD) in rats. This optimal dosage was identified following administration for a duration of four weeks, with dosages ranging from 5 to 30 mg/kg orally^110^. Additionally, naringenin has been consistently administered orally at a dosage of 20 mg/kg in various studies focusing on its hepatoprotective effects^115–117^.

## Evaluation of serum liver function tests

Isolated sera were evaluated for ALT, AST, and alkaline phosphatase (ALP) utilizing colorimetric kits acquired from Biodiagnostics (Giza, Egypt). Additionally, total bilirubin, direct bilirubin, indirect bilirubin, and albumin were measured using a colorimetric method, while lactate dehydrogenase (LDH) was assessed through a kinetic method with kits obtained from EGY-CHEM for Lab Technology (Badr City, Cairo, Egypt). All procedures were performed according to the manufacturers’ instructions.

## Evaluation of hepatic tissue oxidative stress markers

Liver tissue samples were homogenized in phosphate-buffered saline (PBS) and the resulting homogenates were used for the determination of GSH, catalase (CAT), malondialdehyde (MDA), and nitric oxide. These parameters were assessed using commercially available colorimetric kits from Biodiagnostics (Giza, Egypt), in accordance with the manufacturers’ protocols.

## Enzyme linked immunosorbent assay for HMGB1, PI3K, mTOR, TNF-α, TGF-β, and IL-6

Liver tissue samples were homogenized in PBS (pH 7.4). After centrifugation at 3000 rpm for 20 min, the supernatant was collected and analyzed using ELISA for the following markers: HMGB1 (MyBioSource, British Columbia, Canada, Cat# MBS729203), PI3K (MyBioSource, British Columbia, Canada, Cat# MBS260381), mTOR (MyBioSource, British Columbia, Canada, Cat# MBS744326), TNF-α (CUSABIO, Houston, Texas, USA, Cat# CSB E11987r), TGF-β (MyBioSource, British Columbia, Canada, Cat# MBS260302), and IL-6 (R&D Systems, Minneapolis, Minnesota, USA, Cat# R6000B). All assays were performed according to the manufacturers’ instructions. Tissue protein was determined using the biuret method^118^, and data were expressed per milligram of tissue protein.

## qRT-PCR assay for *JAK2* and *STAT3*

To measure *JAK2* and *STAT3* expression, tissue samples were homogenized, and total RNA was extracted using the SV Total RNA Isolation System (Thermo Scientific, USA). The extracted RNA was reverse-transcribed into complementary DNA (cDNA) using the High-Capacity cDNA Reverse Transcription Kit (Thermo Fisher Scientific, USA). The thermal cycling conditions for cDNA synthesis included an initial step at 25 ℃ for 10 min, followed by 37 ℃ for 120 min, 85 ℃ for 5 min, and a final hold at 4℃.

Quantitative real-time PCR (qPCR) was performed using SYBR Green Master Mix detection on Applied Biosystems StepOne™ software (version 3.1). Specific primers for *JAK2* and *STAT3* were used, with β-actin serving as the housekeeping gene for normalization (Table 1). The qPCR cycling protocol included UDG pre-treatment at 50 ℃ for 2 min, initial denaturation at 95 ℃ for 10 min, followed by 40 cycles of denaturation at 95 ℃ for 15 s, and annealing/extension at 60 ℃ for 60 s. Relative quantification was calculated using the 2^−ΔΔCt^ method^119^ and expressed as fold changes relative to the control gene expression levels.

**Table 1:** Primer sequences used for qPCR analysis of JAK2, STAT3, and β-actin.

Gene	Forward primer sequence	Reverse primer sequence
*JAK2*	5′-AGCTCCTCTCCTTGACGACT-3′	5′-GCACGCACTTCGGTAAGAAC-3′
*STAT3*	5′-CAGCAATACCATTGACCTGCC-3′	5′-TTTGGCTGCTTAAGGGGTGG-3′
*β-actin*	5′-CTACGTCGCCCTGGACTTCGAGC-3′	5′-GATGGAGCCGCCGATCCACACGG-3′

## Histopathological and immunohistochemical examinations

Liver tissue samples were fixed in 10% neutral buffered formalin for 72 h, followed by dehydration in graded ethanol concentrations and clearing in xylene. The samples were then embedded in Paraplast embedding medium, and 5 µm-thick serial sections were prepared using a rotary microtome. The sections were mounted on glass slides and stained with hematoxylin and eosin (H&E) for general histological evaluation. Light microscopic examination was performed in a blinded manner by an experienced histologist. All fixation, processing, and staining procedures were carried out in accordance with previously established protocols^120^.

For immunohistochemical (IHC) evaluation, 5 µm-thick paraffin-embedded liver sections were prepared and subjected to deparaffinization and antigen retrieval, followed by treatment with 0.3% hydrogen peroxide for 20 min to block endogenous peroxidase activity. Sections were incubated overnight at 4 ℃ with the following primary antibodies: cleaved caspase-3 (Novus Biologicals, 1:1000), phosphorylated NF-κB p65 (GeneTex, GTX54672, 1:100), MMP-9 (GeneTex, GTX100458, 1:200), and TLR4 (Bioss USA, bs-1021R, 1:200). After washing with PBS, sections were incubated with HRP-conjugated secondary antibodies using the EnVision kit (DAKO) for 20 min, followed by visualization with diaminobenzidine (DAB) and counterstaining with hematoxylin. The stained sections were dehydrated, cleared in xylene, and coverslipped. At least six randomly selected non-overlapping fields per sample were analyzed using the Leica Application Module integrated with a full HD microscopic imaging system (Leica Microsystems GmbH, Germany) to determine the mean relative area percentage of immunopositive hepatocytes for each marker^121^.

### Statistical analysis

The data was statistically analyzed using GraphPad Prism version 9.5.1 Demo (GraphPad Software, San Diego, CA). A one-way analysis of variance (ANOVA), Followed by Tukey’s multiple comparisons test, was employed to evaluate group differences. Results are expressed as mean ± standard deviation (SD), with significance set at *P* < 0.05.

## Supplementary Information

Below is the link to the electronic supplementary material.

Supplementary Material 1

## Data Availability

The datasets generated and/or analyzed during the current study are available from the corresponding author, Mahmoud A. Alkabbani (email: Mahmoud-kabbani@eru.edu.eg), upon reasonable request.
