# Supplementary material for: Protective effects of melatonin and naringenin against acitretin induced hepatotoxicity via modulation of oxidative stress and inflammatory signaling
Source: Sci Rep. 2025 Aug 27;15:31629. doi: 10.1038/s41598-025-16740-9 (PMC12391416; doi:10.1038/s41598-025-16740-9)
Supplement: Supplementary file 1 — Supplementary Material 1 [file 41598_2025_16740_MOESM1_ESM.pdf]

Supplementary Data

Protective effects of melatonin and naringenin against acitretin induced hepatotoxicity via modulation of oxidative stress and inflammatory signaling

Samia S. Sokar[a], Sally E. Abu-Risha[a], Mahmoud Abdelrahman Alkabbani[ab]*, Laila A. Ramadan[b], Alaa E. Elsisi[a]

[a] Department of Pharmacology & Toxicology, Faculty of Pharmacy, Tanta University, Tanta, Egypt.

[b] Department of Pharmacology & Toxicology, Faculty of Pharmacy, Egyptian Russian University, Cairo 11829, Egypt.

* (Corresponding author: Mahmoud Abdelrahman Alkabbani, https://orcid.org/0000-0002-2871-2603, Email: Mahmoud131154@pharm.tanta.edu.eg, Tel: +201030925406)

Preliminary study for Acitretin induced liver injury

Species: Sprague Dawley Rat

Drug: Acitretin

I. Experiment:

CON: Control; 0.5% CMC, Dose: 4 ml/kg every other day

1 D: Acitretin was given in a dose of 10 mg/kg every other day

3 D: Acitretin was given in a dose of 30 mg/kg every other day

6 D: Acitretin was given in a dose of 60 mg/kg every other day

10 D: Acitretin was given in a dose of 100 mg/kg every other day

Rats were sacrificed after 4 weeks.

Assayed Parameters: Serum alanine aminotransferase (ALT), aspartate aminotransferase (AST), alkaline phosphatase (ALP), total bilirubin, direct bilirubin, and indirect bilirubin, and histopathological examination.

II. Results

Table S1. Serum liver function markers

	CON	1 D	3 D	6 D	10 D
**ALT (U/ml)**	20.23 ± 7.15	86.78 ± 11.12^{a}	117.82 ± 8.94^{ab}	160.42 ± 12.08^{abc}	206.74 ± 8.44^{abcd}
**AST (U/ml)**	35.8 ± 7.98	117.02 ± 11.96^{a}	166.33 ± 9.71^{ab}	218.42 ± 6.72^{abc}	281.15 ± 6.89^{abccd}
**ALP (U/ml)**	91.1 ± 8.8	170.05 ± 5.71^{a}	212.9 ± 33.37^{ab}	296.23 ± 7.91^{abc}	358.62 ± 14.32^{abcd}
**Total bilirubin (mg/dl)**	0.44 ± 0.07	1.19 ± 0.09^{a}	1.8 ± 0.04^{ab}	2.05 ± 0.05^{abc}	2.28 ± 0.11^{abcd}
**Direct bilirubin (mg/dl)**	0.18 ± 0.05	0.4 ± 0.07^{a}	0.53 ± 0.04^{ab}	0.65 ± 0.04^{abc}	0.76 ± 0.07^{abc}
**Indirect bilirubin (mg/dl)**	0.27 ± 0.04	0.79 ± 0.04^{a}	1.27 ± 0.04^{ab}	1.4 ± 0.08^{ab}	1.52 ± 0.11^{abc}

Data (mean ± SD) were subjected to one-way ANOVA, subsequently followed by Tukey's multiple comparisons. [a] Significantly different from CON group at $p < 0.05$, [b] Significantly different from 1 D group at $p < 0.05$, [c] Significantly different from 3 D group at $p < 0.05$, [d] Significantly different from 6 D group at $p < 0.05$.

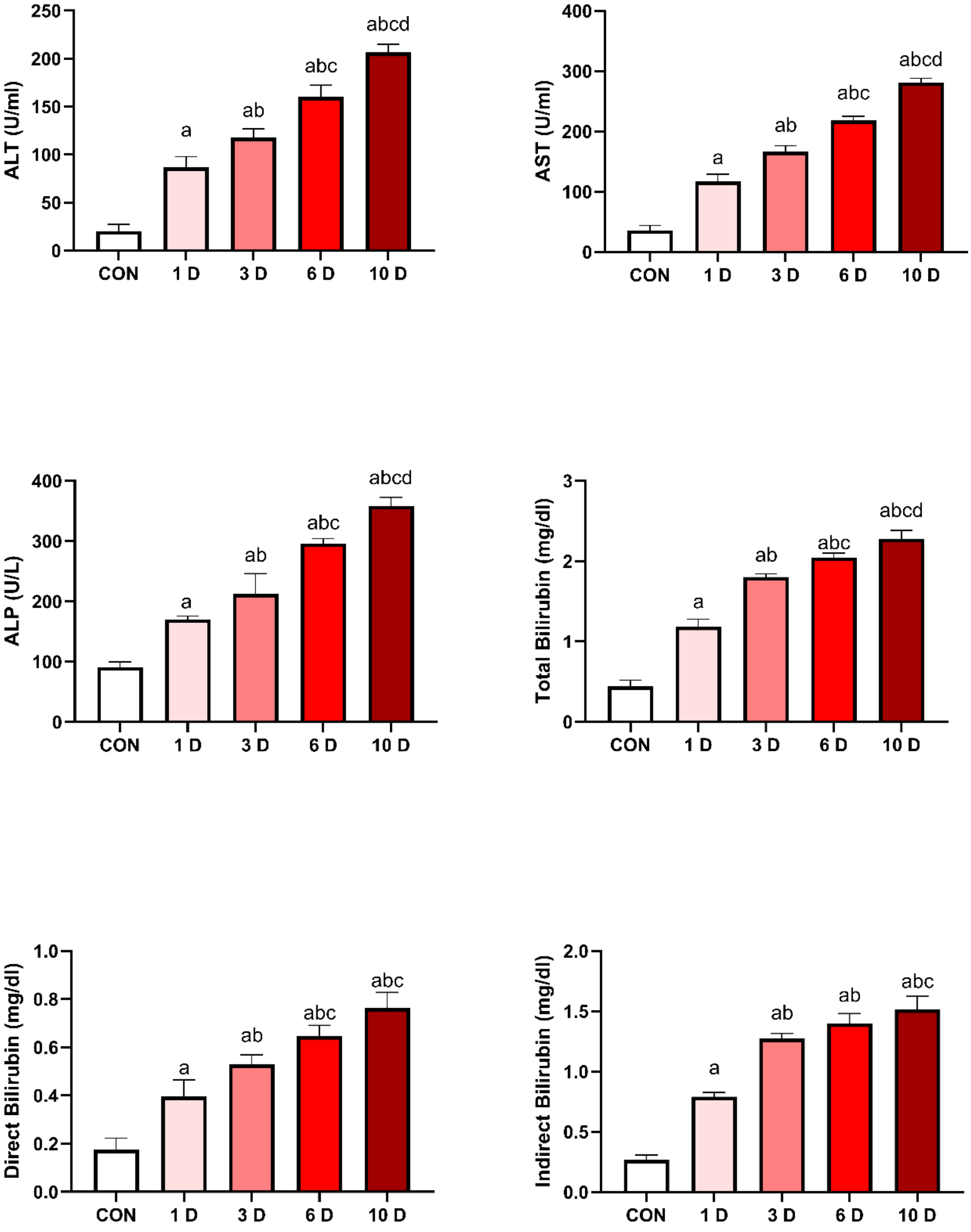

Figure S1. Serum liver function markers. Data (mean ± SD) were subjected to one-way ANOVA, subsequently followed by Tukey's multiple comparisons. [a] Significantly different from CON group at $p < 0.05$, [b] Significantly different from 1 D group at $p < 0.05$, [c] Significantly different from 3 D group at $p < 0.05$, [d] Significantly different from 6 D group at $p < 0.05$.

Histopathological examination

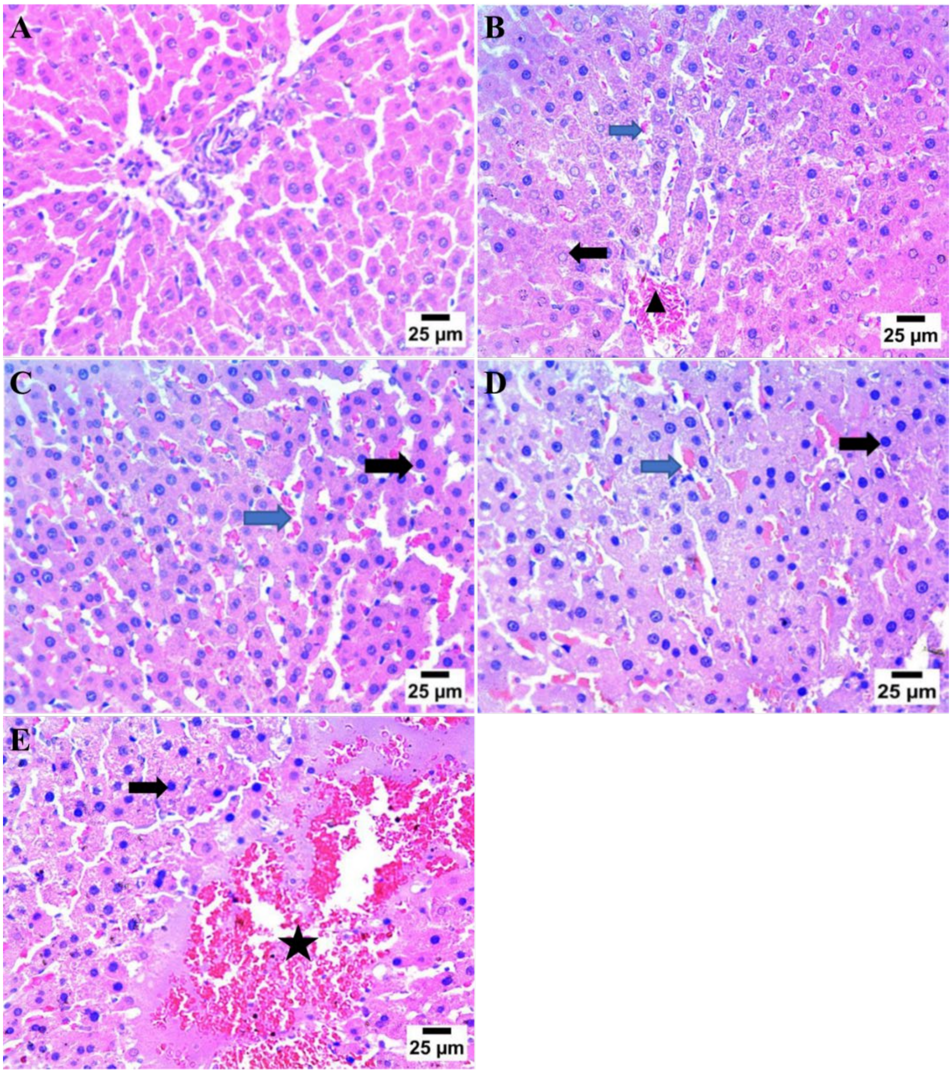

Figure S2. Photomicrographs of Hematoxylin and Eosin (H&E) stained liver sections show normal histological architecture of hepatocytes and portal area in the control (CON) group (A). In the 1 D group (B), central vein congestion (arrowhead), dilated hepatic sinusoids engorged with blood (blue arrow), and necrobiotic changes in some hepatocytes (black arrow) are observed. The 3 D group (C) exhibits sinusoidal dilatation with blood engorgement (blue arrow) and marked nuclear pyknosis in hepatocytes (black arrow). The 6 D group (D) shows extensive nuclear pyknosis in all hepatocytes (black arrow) along with dilated sinusoids engorged with blood (blue arrow). In the 10 D group (E), severe hemorrhage between hepatocytes (star) and severe nuclear pyknosis in all hepatocytes (arrow) are evident.
